# Application of Capillary Electromigration Methods in the Analysis of Textile Dyes—Review

**DOI:** 10.3390/molecules27092767

**Published:** 2022-04-26

**Authors:** Anna Sałdan, Małgorzata Król, Michał Woźniakiewicz, Paweł Kościelniak

**Affiliations:** Laboratory for Forensic Chemistry, Department of Analytical Chemistry, Faculty of Chemistry, Jagiellonian University in Krakow, 30-387 Kraków, Poland; anna.saldan@doctoral.uj.edu.pl (A.S.); michal.wozniakiewicz@uj.edu.pl (M.W.); pawel.koscielniak@uj.edu.pl (P.K.)

**Keywords:** textile dyes, single fiber, capillary electrophoresis, capillary electromigration methods

## Abstract

Fiber traces are one of (micro)traces that can be found at a crime scene. They are easily transferable and, like other forms of evidence, can provide a link between a suspect and a victim. The main purpose of this review is to present methods developed to examine textile dyes extracted for forensic purposes using different capillary electromigration methods (CEMs). Scientific papers, mainly from the 20th century, provide reliable methods for the separation of water-soluble dyes. However, dyes insoluble in aqueous solutions have been and still are a challenge. Another problem is the sensitivity of the developed methods, which is, in most cases, insufficient for forensic examination of dyes extracted from a single fiber preserved at the crime scene. Although the methodologies already developed and presented in this review have the potential to be applied in a comparative analysis of textile dye traces, there seems to be a lot of work to be conducted. Some ideas on how to resolve these problems are presented and discussed in the article.

## 1. Introduction

### 1.1. Fibers as Forensic Traces

Among many traces that can be found at the scene of a crime, microfibers undoubtedly play an important role. Despite their small size, these fibers can be a source of essential information, which often turns out to be a valuable guide in evidence proceedings. The main goal of the research is to determine whether the microfibers found at the crime scene may originate from comparative material secured during the investigation (for example, from a suspect’s clothing).

The fiber examination can be performed in two ways. The first approach covers group identification, consisting in determining the type of fiber, classifying it, and, possibly, specifying the product from which it may come. The second type is comparative research. Using various methods, they enable to obtain information about the physicochemical properties of fibers preserved from both sources. Then, the data obtained for both materials are compared, and consequently, with a certain probability, the common origin of the investigated fibers is excluded or confirmed [1].

The chemical composition of the fibers or the type of finishing agents (finishes) used may be an important identifying factor. Analyzed fibers may also show signs of mechanical or thermal damage, as well as contain contaminants in the form of biological fluids or trace amounts of substances [2,3].

Microtraces in the form of fibers are most often used as evidence in cases related to batteries, homicides, or sexual offences. Then, fragments of fibers from the victim’s clothing are searched for on the attacker’s clothing and contrariwise, as well as in the matter under the fingernails. It also happens that fibers are used as evidence in hit-and-run accidents involving pedestrians. In such cases, the traces are secured from parts of the car body and chassis. If the offence was committed with the use of a specific tool, e.g., a knife or a screwdriver, it is also possible to reveal microfibers from the victim’s clothing on the tool.

Single fibers secured at the crime scene most often have a diameter of 15–25 μm and a length of 1–3 mm. Due to their small size, they are imperceptible without the use of appropriate equipment. To protect them, a transparent, nonadhesive foil or tape is used. These materials are usually supplied with an appropriate substrate that protects the evidence against contamination. An important factor concerning the traces in question is the time elapsed from the incident to the preservation of the microtraces. According to the literature, even 80% of the transferred fibers are lost in the first four hours after the transfer in contact [4].

### 1.2. Characteristics of the Fibers

Fibers are associated primarily with clothing, but they can also be found in decorative home furnishings such as carpets, curtains, and upholstery, as well as in means of transport, where they appear on seat upholstery, seat belts, and carpets. Fibers are defined as flexible and strong objects, the length of which significantly exceeds the dimensions of the cross-section [5]. Length, thickness, strength, elasticity, elongation, and hygroscopicity are significant features allowing one to characterize the properties of a given fiber. In most cases, the fibers are made of large-sized organic molecules called fiber-forming polymers. However, there are also fibers that are built from low-molecular inorganic compounds. These include, among others, glass and carbon fibers.

The easiest way to divide fibers into two groups is: natural and chemical. Natural fibers, as the name suggests, are fibers that occur in nature, while chemical fibers are produced by man. Natural fibers can be of plant, animal, or mineral origin. Among chemical fibers, one can distinguish artificial fibers, i.e., those that were made of polymers of natural origin using appropriate techniques, and synthetic fibers, which are obtained from fiber-forming polymers.

Statistical data show that synthetic fibers are currently used more often than natural fibers. In 2019, polyester fibers accounted for 52.2% while cotton only for 23.2% of global fiber production (~111 million metric tons) [6,7]. However, during the assessment of the significance of a fiber as forensic evidence, not only its type, but also its color is taken into account [6]. There are categories of fibers that are very popular and widespread in the environment, e.g., white cotton fibers, which are therefore a trace of negligible importance from the point of view of the forensic evaluation of evidence. Therefore, very often the disclosure of less common types of synthetic fibers of unique color may increase the significance of such a trace.

### 1.3. Characteristics of Coloring Substances

An important element of the textile production process is the dyeing of the material. Dyes or pigments are used to give the fabric the right color. These are compounds that absorb and reflect visible light at specific wavelengths. Dyes can interact with the fibers of dyed fabrics. These can be physical interactions (ionic bonds, van der Waals forces, and hydrogen bonds) or chemical interactions, namely covalent bonds [8]. On the other hand, pigments, unlike dyes, do not exhibit an affinity for fabrics, but only penetrate between their fibers. In most cases, the dyeing process uses multiple dyes.

The most popular source of information on colorants used in industry and commerce is the International Color Index (C.I.). Currently, it is an international database maintained by SDC (Society of Dyers and Colorists) and AATCC (American Association of Textile Chemists and Colorists). All dyes have an individual generic CIGN name (related to an application class) and the CICN number (related to the chemical structure). At present, the database contains over 13,000 chemical structures, and 37,000 trade names of available coloring substances [9].

**Figure 1:**
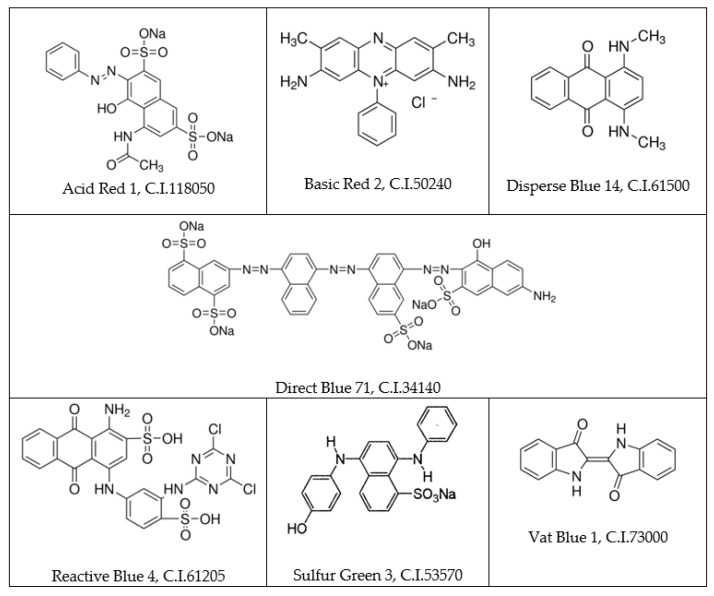
The exemplary molecular structure of dye form various groups: acidic, basic, direct, disperse, reactive, sulfur, and vat.

Due to the method of application, dyes can be divided into, among others, acid, basic, direct, disperse, reactive, sulfur, and vat [10]. Figure 1 shows the chemical structure of an exemplary representative of each mentioned group. Table 1 shows the groups of dyes along with the types of fibers they dye. This way of classifying dyes is related to the nature of the interactions between the dye and the dyed fiber. Acid dyes interact with fiber through ionic bonds (similarly to basic dyes) and hydrophobic interactions. Direct dyes bind to fibers by hydrogen bonds and Van der Waals forces, and reactive dyes by covalent bonds. In turn, disperse dyes, through the stage of molecular dispersion, diffuse deep into the dyed fibers. The International Color Index distinguishes 19 classes related to the use of dyes. The group of acid dyes is the most numerous, followed by direct and disperse dyes [11].

The second criterion for the classification of dyes is based on the differences in the chemical structure of the chromophores within the molecule. The International Color Index distinguishes 25 structural classes [10,11]. Among them, there are groups such as azo, anthraquinone, indigoid, arylmethane, phthalocyanine, methine, and other dyes. Most compounds are found in the groups of azo and anthraquinone dyes. The first of them are characterized by a lower price and greater color efficiency. On the other hand, the latter are more resistant to light and more bright than azo dyes.

## 2. A General Outline of Methods Used in Forensic Examination of Fibers

When testing microfibers, non-destructive methods are used at first. They allow the trace to be analyzed again or examined using a different method afterwards. Only when non-destructive methods do not provide sufficient information about the trace under investigation, the validity of the use of the destructive method is considered.

The first step in the analysis of the collected fibers is the assessment of their characteristic structural features. Based on these characteristics, the fiber can be pre-assigned to a specific type. For this purpose, a longitudinal view of the fibers, or their cross-section, is observed under a microscope. These observations make it possible to determine the type of fibers (especially natural ones) and their diameter. Further data can be obtained using a polarized light microscope. Such a test can provide information on the distribution of crystalline and amorphous particles in the examined fiber. As a result of this, it is possible to perform an initial classification of fibers (mainly synthetic).

Infrared (IR) spectroscopy is a frequently used technique for identifying the chemical compositions of the fibers. In turn, the Raman spectroscopy technique is used for the analysis of dyes and finishing agents. The advantage of these techniques is the possibility of combining spectrometers with microscopes, which enables the analysis of individual fiber fragments. In order to determine the color, one can also use a fluorescence microscope or use the technique of microspectrophotometry (MSP) in the ultraviolet and visible range (UV-Vis) [1,12]. The fluorescence microscopy is recognized as a powerful technique merging the structural analysis of fibers with the identification of dyes and pigments. The main advantage of these techniques is the fact that they do not damage the analyzed samples. Moreover, the analysis requires a small amount of sample which does not require complicated preparation [6].

Usually, a mixture of several dyes is used to obtain the required hue of textile that makes the analysis more complex [4]. The analytical investigation is additionally hindered by the fact that the amount of dye in the microfibers secured at the crime scene is typically very small. For example, 5–10 mm long fiber may contain 2 to 200 ng of dye [6]. This requires searching for identification methods characterized by high sensitivity. Such a need leads the examiners to look for chromatographic methods, such as the high performance liquid chromatography (HPLC) or ultrahigh performance liquid chromatography (UHPLC) hyphenated with a wide range of possible detectors [6,8,13,14]. The high sensitivity of instrumental chromatographic techniques recompenses the fact that they are destructive to the sample. These techniques also require appropriate preparation of the samples analyzed, mainly solid-liquid extraction (SLE) assisted by high temperature, shaking, or ultrasonication [13,15,16,17,18,19].

Thus, many effective methods have been developed and are constantly being improved for forensic comparative and identification examination of fibers. This review, due to volume limitations, does not present all of them in-depth, details can be found in the following articles [4,6,20,21,22]. Capillary electromigration methods (CEMs), due to their advantages, in particular the small amount of sample required, seem to be the methods of choice for this purpose [23,24]. Although CEMs require the extraction of dyes, they can be considered as semidestructive, i.e., destroying the analyzed object only to a limited extent). Highly efficient and selective separation of various types of analytes in one analysis can greatly increase the degree of discrimination of similar fibers.

The multitude of available CEMs techniques gives great opportunities to control the selectivity of method depending on the needs and type of application. Significant improvements in selectivity can be achieved by small modifications of the BGE composition or the separation conditions such as temperature or voltage, without the need to interfere with the instrument components. Furthermore, CEMs provides the possibility of using a variety of techniques enabling the concentration of analytes inside the capillary, using stacking techniques. Their advantage shows up in the sensitivity increase and the simplicity as such improvement does not require any additional equipment.

Depending on the structure and the related properties of the analyzed compounds, it is possible to select the most appropriate detection method from a number of available options: direct or indirect UV-Vis absorption, fluorescence, or laser induced fluorescence, amperometry, conductivity, and mass spectrometry.

Considering the practical aspects related to the application of the discussed separation methods, the advantage of CEMs over HPLC is increased cost effectiveness in terms of operation and reagents consumption, making CEMs excellent examples for green analytical chemistry methods. Taking into account the global efforts on improvements of analytical techniques in terms of ecofriendliness, we decided to focus this review solely on the capillary electromigration methods. While the methodologies already developed and presented in this review have the potential to be used in the comparative analysis of textile dye traces, it seems that CEMs offer many other possibilities that can be further exploited. The authors have attempted to discuss major of them in this review article.

## 3. Application of Capillary Electromigration Methods to the Analysis of Textile Dyes

While reviewing the articles that reported the examination of textile dyes with the use of CEMs, it draws attention that this topic was intensively explored in the 90s. At the beginning of the 21st century, individual reports appeared, after 2010 only one publication can be identified. So, the question arises whether this is since everything that could have been achieved has already been obtained, or if some areas of textile dyes examination have been and still are a challenge for analytical chemists. Or maybe there are groups of textile dyes that, for some reason, cannot be analyzed using CEMs. The authors of this review will try to present and discuss the current state of knowledge regarding the analyses of textile dyes using CEMs, the achievements of scientists and the confirmed possibilities of these techniques, as well as aspects that still need to be refined.

### 3.1. Capillary Electromigration Analysis of Acid Dyes

Acid dyes are most often used to dye polyamide and wool fibers under acidic conditions. They contain a significant number of hydrophilic groups in their structure. They are in anionic form, which makes it easy to separate them using the simplest of CEMs called capillary zone electrophoresis (CZE) or simply capillary electrophoresis (CE). Since these are negatively charged compounds, using fused-silica capillary, pH of the background electrolyte (BGE) should be higher than p*K*_a_ of the surface silanol groups and the magnitude of the electroosmotic flow (EOF) must be greater than the electrophoretic mobility of these particles to force them to move towards the cathode. Increasing the EOF can be obtained by increasing the voltage, decreasing the concentration and thus the ionic strength of the BGE or increasing the ionization of the capillary walls by increasing the pH of the BGE. In their 1992 work, Croft and Hinks used the third of these approaches [25]. They used a BGE containing 10 mmol·L^−1^ of potassium dihydrogen phosphate with a pH = 9. As a result, they separated four acid dyes together with three non-colored intermediates in their synthesis within 30 min. In the course of the work, researchers observed that at higher pH values, the compounds migrated quickly enough that their separation deteriorated. On the other hand, at lower pH values, the separation took a very long time, even about 80 min.

Another example of the use of CZE for the analysis of this class of dyes was presented by Sirén and Sulkava in 1995 [26]. They analyzed black dyes extracted from woolen fibers. In their research, they used a BGE containing 0.14 mol·L^−1^ (3-cyclohexylamino)-1-propanesulfonic acid (CAPS), pH = 10.8. They used a UV-Vis detector established at a wavelength of 254 nm. The developed method allowed them to separate six acid dyes with good repeatability in a time not exceeding 16 min.

In the case of analytes that are weak acids, their degree of dissociation and thus electrophoretic mobility can be influenced by changing the pH of the BGE. It is then possible to select the optimal pH for the separation of the examined compounds. However, for substances that are strong electrolytes, the appropriate pH of the buffer is not always sufficient and therefore it is necessary to use other separation methods. One of the possible solutions to this problem was presented by Blatny et al. [27], who introduced soluble polymers—polyethylene glycol (PEG) and polyvinylpyrrolidone (PVP)—to the BGE. They may introduce lipophilic interactions with the analytes, analogously to the case of the reverse phase chromatography. In CE, these polymers dispersed in BGE form so called pseudostationary phases. The more aromatic rings a dye molecule has, the stronger it interacts with the polymer, which extends its migration time. The BGE that allowed the separation to be performed consisted of 0.5% (m/v) PEG, 0.01% (m/v) PVP and 1,3-bis[tris(hydroxymethyl)methylamino]propane (BIS-TRIS-propane) with pH = 6.5.

In 2009, the possibility of using CZE for the separation of acid dyes extracted from the nylon fibers was presented. The extraction efficiency for three subclasses of acid dyes (anthraquinone, azo, and metal complex) was verified in combinatorial experiments using three-component mixture designs varied three solvents (pyridine/ammonia/water) at different temperatures for various durations. The composition of the BGE was also optimized to ensure that the dye molecules are in the form of anions during the separation. Acid dyes are soluble in solvents of pH above their p*K*_a_, therefore, in this case, pH of the BGE was equal 9.3. Apart from the signals from dyes, the researchers reported other unidentified peaks on obtained electropherograms. As they were found in many extracts, it was hypothesized that peaks either come from residuals associated with the dyeing process of fabrics or are contaminants of dyes still from the stage of their synthesis. In combination with appropriate extraction parameters (pyridine/ammonia, 1:1, *v*/*v*; 100 °C for 15 to 30 min), the method presented in the research can be used for forensic purposes [28].

The same background electrolyte was also successfully used in an analysis of a mixture of four acid dyes extracted from nylon fibers and in the separation of a mixture of acid, direct, and reactive dyes, extracted from cotton and nylon fibers. In their work, Morgan et al. used a diode array detector that had sufficient sensitivity for the efficient analysis of extracts collected from 1 cm long fiber [29].

An interesting example of the use of CZE-MS was the study of the degradation mechanism of selected acid dyes published in 2008 [30]. Separation of dye degradation products was carried out in a buffer containing 0.1 mol·L^−1^ ammonium acetate, pH = 9. The use of an electrospray ion source coupled to the mass spectrometer allowed one to track the decay paths of the two acid dyes Acid Orange 7 and Acid Orange 8.

Typically, CEMs performed in aqueous background electrolytes are suitable for the separation of charged molecules. However, in such an environment, hydrophobic interactions between the capillary walls and some colored compounds may arise, which distort the shape of the obtained peaks. In such cases, non-aqueous capillary electrophoresis (NACE) may be an effective alternative [31]. The p*K*_a_ values of the analytes may be shifted in organic solvents compared to aqueous solutions. Consequently, an appropriate selection of organic solvents and their proportions can answer many problems related to the separation of target compounds. The NACE method was used by Peláez-Cid et al., to separate the acid and basic textile dyes extracted from the surface water samples using solid phase extraction (SPE, more details in Table 2). Researchers used electrochemical (cyclic voltammetry) detection, which made it possible to achieve significantly lower LOD values (0.1–0.7 μg·mL^−1^) than in the case of UV detection (0.7–2.2 μg·mL^−1^). The composition of the BGE suitable for the simultaneous separation of acid and basic dyes and compatible with both the detection systems is presented in Table 2. The developed method can be used to determine the content of textile dyes in the surface water, thus can be used for environmental monitoring [31].

Compounds with the same charge are separated mainly due to differences in the charge to mass (or more exactly hydrodynamic size) ratio. Therefore, in the case of basic background electrolytes and CE operating in normal polarization, acid dyes should migrate through the detection window in order of decreasing the mass-to-charge ratio. One should notice that such general assumption is not always experimentally confirmed, because the apparent electrophoretic mobility is also affected by other factors, such as the presence of functional groups, interactions with the capillary wall, hydrophobic interactions, solvation, and viscosity [25]. In the case where we are dealing with compounds of the same charge and very similar structure and relative molecular mass (e.g., isomers), the described interactions may not ensure the desired separation of the substance. Burkinshaw, Hinks, and Lewis faced this situation in 1993. In their research, two acid textile dyes (as a standard solution) were analyzed using 10 mmol·L^−1^ potassium dihydrogen phosphate buffer at pH = 9 and which was found unsuccessful in separation capacity. Providing additional interactions, namely hydrophobic ones with pseudostationary micellar phase in the micellar electrokinetic capillary chromatography (MEKC), enabled the separation of the investigated compounds. For this purpose, they used a BGE which composition is listed in detail in Table 2 [32]. The next steps of these examinations leading to the successful separation of also neutral dyes, and the number of charged and uncharged, water-soluble, and water-insoluble dye intermediates will be presented in the section on disperse dyes.

The use of MEKC also turned out to be a key in the analysis of the commercial dye Acid Black 194. This dye forms three types of complexes with metals that show higher toxicity to aquatic organisms. The method was developed to test surface waters for the presence of acid dyes coming from wastewater disposed of after the dyeing process [33]. The researchers wanted to separate these three isomeric complex compounds, pure ligand, and impurities that were components of a commercially available Acid Black 194 dye. The isolation of the metal complex dyes was carried out by flash chromatography using a direct-phase silica gel column (35–70 μm, I.D. 5 cm, length 20 cm), mobile phase flow 2.5 cm·min^−1^ and the eluent consisted of AcOEt: MeOH: AcOH (8:1:1, *v*/*v*/*v*). The preparation of the dye stock solutions directly before injection into instrument is presented in Table 2. To this end, they used many analytical techniques, including reverse-phase HPLC and CZE with the use of various separation buffers. They achieved satisfactory results only after using MEKC with a separation buffer containing 10 mmol·L^−1^ SDS in 25 mmol·L^−1^ sodium tetraborate and pH 9.3. The 12 components of the samples were separated from each other within 8 min.

The instrumental conditions used in the above-mentioned examples of acid dye separation are shown in Table 2.

### 3.2. Capillary Electromigration Methods in the Analysis of Basic Dyes

Basic dyes are used in the dyeing of mainly polyacrylonitrile and acrylic fibers. Basic dye molecules are in the form of cations, and therefore, as in the case of acid dyes, the CZE technique can be used for their separation. As a result of the positive charge, these compounds move toward the cathode according to their electrophoretic mobility. In BGEs of pH higher the p*K*_a_ of silica, the electroosmotic flow increases the speed of migration of ions along the capillary, which significantly reduces the analysis time, but may also deteriorate the quality of separation. Theoretically, the lower the mass-to-charge ratio of cation, the shorter the migration time of a specific compound is. However, in practice, this rule of thumb may be affected due to the presence of other factors, similarly to the case of acid dyes.

The separation of four basic dyes along with three non-colored compounds with a positive charge was carried out by Croft and Hinks, using a BGE containing 20 mmol·L^−1^ of citric acid at pH = 4.5 [25]. They observed that at a buffer pH above 6, a peak corresponding to dyes did not appear on the electropherogram even with longer measurement times. Since increasing the pH of the buffer causes a negative charge to appear on the capillary walls, ionic bonding between positively charged basic dye molecules and the -SiO^−^ groups may have occurred. Another possible explanation is that with the increase of pH, the number of dissociated acid molecules increases, which can form complexes with dyes and precipitate inside the capillary. This study shows how important it is to choose the right pH of the BGE and how many factors should be taken into account in this fragile equilibrium.

For some basic dyes, even the use of a suitably low pH is not sufficient to prevent cations adsorption to the inner capillary wall. In their studies, Blatny et al. [34] observed that even at pH = 3, ionization of silanol groups may appear, providing on the weak electroosmotic flow. However, with subsequent measurements, the flow rate decreased, most likely because of the adsorption of cationic dyes on the inner capillary surface. The variation of the EOF velocity deteriorates the repeatability of migration time and further—the peak area. This issue was mitigated by the addition of PVP forming as a pseudostationary phase, which improved the quality of separation and repeatability due to the reduction of the EOF.

In 2009, Stefan et al. [35] presented the possibility of using CE in the analysis of basic dyes. They separated seven dyes using a background electrolyte containing 45 mmol·L^−1^ ammonium acetate in the mixture ACN:H_2_O (60:40, *v*/*v*) and pH = 4.7, the low pH of which guaranteed an appropriate degree of ionization of the dyes. In turn, the addition of an organic solvent kept the current at a sufficiently low level during the measurements. This BGE was then used to separate the dyes extracted from the acrylic fibers. It was found that for extracts of fiber with a length of not less than 1 cm, a diode array detector provides enough sensitivity. On the other hand, for shorter fibers, a more sensitive detector should be used (e.g., ESI-QTOF-MS).

As mentioned earlier, CEM in the NACE mode can be successfully used in the analysis of basic dyes. In 2008, the separation of a mixture containing five basic dyes and two acid dyes was reported. In this study, a BGE consisting of 1 mol·L^−1^ acetic acid and 10 mmol·L^−1^ ammonium acetate in the ACN:MeOH mixture (75:25, *v*/*v*) was applied [31]. The medium baseline separations could be achieved for all analyzed dye compounds with limits of detection (LOD) in the range of 0.1–0.7 μg·mL^−1^. The proposed analytical protocol was successfully applied to various water samples (spiked with textile dyes), including river and lake water.

The instrumental conditions used in the above-mentioned CEMs employed in the separation of basic dyes are presented in Table 2.

### 3.3. Capillary Electromigration Analysis of Reactive Dyes

Reactive dyes are water-soluble compounds. They are used to dye wool, cotton, and cellulose fibers. In the process of dying, they form covalent bonds with the functional groups of fibers (e.g., the cellulosic hydroxyl groups [42] or the amino group of silk fibroin [43]), therefore they are resistant to rinsing during washing [4]. Although there are differences in the mechanism of the dying process, reactive dye molecules like acid dyes are anions, so the separation process may be performed likewise.

In 1992, Croft and Hinks conducted examinations to separate a mixture of five reactive dyes (solution with a concentration of 1 g·L^−1^) using HPLC and CZE [25]. The HPLC method was unsuccessful in the separation of two out of five components of the mixture, while CZE was found to be efficient. The authors managed to separate all the components within 14 min.

Similar studies comparing HPLC with CZE were conducted in 1999 [36]. In the case of HPLC, it was not possible to separate the two dyes, while CZE enabled the separation of all five components of the mixture. In this study, a buffer containing 10 mmol·L^−1^ of sodium tetraborate and 6 mmol·L^−1^ of potassium dihydrogen phosphate at pH 8.25 was utilized. What is worth noting is a much smaller sample volume injected in CZE (5 nL in CZE and 10 μL by HPLC). The cost-effectiveness of the CEM was also emphasized. On the other hand, HPLC was characterized by better repeatability than CZE [36].

CZE was also used to analyze extracts of black reactive dyes from cotton fibers. For the separation, a buffer containing 0.14 mol·L^−1^ CAPS at pH 10.8 was used, the same that had been previously used in the analysis of acid dyes. The method was found to be very effective as the separation of eight dyes was performed in less than 10 min [26].

The addition of an organic solvent to the buffer has a significant influence on the separation process. Organic modifiers change the viscosity, reduce the conductivity of the BGE, change the EOF, and may also increase the solubility of the analytes. The effect of the acetonitrile content in BGE was investigated by Farry et al. [37], who separated a mixture of three anionic reactive dyes and one cationic basic dye dissolved in pure water. The increase in the ACN content in the range of 0–25% resulted in a reduction of the EOF, followed by longer migration times but also improved resolution and peak shape (the observed signals were sharper). Above 15% ACN, some peaks were noticed as suppressed, therefore a buffer containing 10% ACN and 20 mmol·L^−1^ sodium tetraborate pH = 9.26 was chosen as the optimum.

In 2009, Dockery et al. investigated the use of CZE in the analysis of reactive dyes extracts obtained from cotton fibers [38]. The main goal of this study was to develop the dye microextraction with the use of solvents and conditions which were compatible with the subsequent forensic characterization of extracted dyes by capillary electrophoresis with UV/Vis diode array detection. Of the extraction methods tested, the use of a monocomponent solvent of 1.5% aqueous sodium hydroxide in combination with purification of the extract using solid-phase reverse phase extraction, gave extracts that were relatively contaminant-free, suitable for subsequent capillary electromigration analysis. Using the optimal BGE of pH = 9.3 (details in Table 2), it was possible to separate the mixture of 12 dyes in less than 15 min.

Reactive dyes as anionic compounds are characterized, in capillary electromigration analysis, by relatively long migration times. This is due to their electrophoretic mobility, which is directed towards the anode, i.e., opposite to the direction of the electroosmotic flow. The longer the migration times of the analytes, the more difficult it is to obtain good repeatability of the analyses, therefore, if possible, the aim is to shorten the migration times of the tested compounds. Oxparing, Smyth, and Marchant presented an interesting way to shorten the analysis time [39]. Reversing the polarity of the electrodes changes the elution order of the compounds in the capillary in such a way that anionic compounds are observed first, then neutral, and finally cationic. The use of a buffer containing 0.05 mol·L^−1^ citric acid and 10% ACN (pH = 3.25) prevented the -SiOH groups (present at the inner capillary wall) from ionizing, limiting zeta-potential and thus excluding EOF. Using this approach allowed researchers to separate six dyes (details regarding sample preparation in Table 2) within 12 min of analysis.

At this point, it is also worth noticing the essential influence of the pH of the BGE on ionization, and what is related to it, the solubility and the electrophoretic mobility of the analyzed substances. The dissociation of acidic or basic groups varies according to the p*K*_a_ of the functional groups concerned. For instance, in the case of Remazol Black B the measured p*K*_a_ values, according to the literature [44], are close to 3.8 and 6.9. Assuming that the p*K*_a_ values of the sodium sulfonate groups which exhibit in dye molecule are known to be very low (<1). Plus, at the same time, the p*K*_a_ value of 3.8 is close to the p*K*_a_ of aniline (i.e., 4.6) so it can be attributed to the –NH_2_ groups of the dye molecules. It can be concluded that at pH < 3.8 and > the p*K*_a_ of the sulfonate groups, the dye molecules are in their anionic forms and have obtained the highest negative charge. This explains their fast migration times under the separation conditions proposed above.

The instrumental conditions used in the above-mentioned examples of separation of reactive dyes are shown in Table 2.

### 3.4. Capillary Electromigration Methods in the Analysis of Vat Dyes

Vat dyes, such as reactive and direct dyes, are used to dye cellulose fibers. The reduced (leuco-) form of these dyes is water-soluble. The vat dye particles are oxidized inside the fibers to a water-insoluble form during the dyeing process.

As CEMs require that the analytes be soluble in the separation buffer, the dyes must be converted to a water-soluble form prior to analysis. An alternative is to use a non-aqueous or microemulsion BGE with the use of an organic solvent/solvents in which the examined dyes are soluble. Dockery et al. [38] faced this problem in their work. They verified different approaches, but the first-mentioned above method was found a more universal solution. The addition of a reducing reagent (Na_2_S_2_O_4_) to the BGE and the sample solution (vat dye mixture) allowed the analysis in a water-based environment. However, at this point, it should be highlighted that vat dyes are a group of substances that differ significantly in terms of chemistry. Let us remind, they are classified in this group due to the dyeing method, not on the basis of properties. This group includes dyes, e.g., indigoid, anthraquinone or violanthrone, which have completely different properties in terms of solubility in certain organic solvents. They are also characterized by the different possibilities of reduction or derivatization to increase solubility. Therefore, it is very difficult to find a universal extraction method as well as a separation protocol. Taking into account these issues, it becomes clear why the results of the separation of vat dyes presented in the above-mentioned article [38] are not spectacular. The authors listed a 12-item collection of vat dyes to be analyzed. However, only moderate success has been achieved. They showed the separation result for only three dyes, not explaining why the number of dyes analyzed was limited. Moreover, the presented electrophoretic profile is characterized by poor resolution and an elevated baseline. In addition, while reading the article, one may come across some incoherencies and understatements that make it difficult to reproduce the presented method. Therefore, it is difficult to assess the suitability of the proposed method in the case of vat dyes analyses.

It was as early as 1994 when the possibilities of using capillary electrophoresis-electrospray ionization-mass spectrometry (CE/ESI-MS) for the separation of three vat dyes with similar structures were presented. Very important information, in this case, concerns the solubility of analyzed compounds. In this paper, the authors successfully attempted to separate colorants soluble in water—the mixture of sulfonated derivatives of indigoid vat dyes (Vat blue 1, Vat blue 2, and Vat blue 3). Vat dyes are, by definition, considered water insoluble, so the authors mislead the readers by simply calling soluble derivatives ‘vat dyes’. The BGE containing 10 mmol·L^−1^ ammonium acetate of a pH = 10 with the addition of 12.5% ACN was proposed as optimal [40].

The instrumental conditions used in the above-mentioned examples of vat dye separation are shown in Table 2.

### 3.5. Capillary Electromigration Methods in the Analysis of Direct Dyes

Direct dyes are anionic compounds, nearly all are azo dyes easily soluble in water. The other, less common, chromophoric structures are as follows: stilbene, phthalocyanine, dioxazine, and smaller chemical classes such as formazan, anthraquinone, quinolone, and thiazole [45]. As the name suggests, these dyes are, in the presence of an alkaline electrolyte, directly deposited on the dyed fibers so their wash-fastness performance is only moderate. They are most often used in dyeing cotton and other cellulose fibers [4].

Due to the negative charge, the molecules of direct dyes behave during separation similarly to acid and reactive dyes. The use of CZE for the analysis of a mixture of seven direct dyes was presented in 2009 by Dockery et al. [38]. They successfully used a BGE containing 5 mmol·L^−1^ ammonium acetate in the ACN:H_2_O (40:60, *v*/*v*) of a pH = 9.3 to perform the separation.

A BGE with a similar composition, but containing threefold more ammonium acetate, was successfully used to separate a mixture of 14 anionic dye standards (dissolved in water) from different classes, i.e., 5 acid, 5 reactive, and 4 direct ones [29].

The instrumental conditions used in the above-mentioned examples of direct dye separation are shown in Table 2.

### 3.6. Capillary Electromigration Analysis of Disperse Dyes

When selecting the variant of electrophoresis, which will enable the separation of disperse dyes, it should be remembered that they are neutral compounds. For this reason, it is not possible to use the CZE technique, which is commonly selected and widely described in the literature in the case of charged dyes. In addition, disperse dyes are poorly soluble in water, which excludes the possibility of using a pure water-based BGE and necessitates looking for other solutions.

One possibility was described in 2007 by Morgan et al. [29]. They investigated disperse dyes extracted from polyester fibers. The separation was achieved thanks to the NACE technique, using a BGE based on a mixture of acetonitrile and methanol (75:25, *v*/*v*). Additionally, the BGE contained 80 mmol·L^−1^ of ammonium acetate, and its pH* = 9 (pH*—pH measured in organic solvents). Replacing the water with organic solvents solved the problem of poor solubility of the analytes in the separation buffer.

It is not always necessary to eliminate water from the buffer composition. In some cases, the addition of only a certain amount of organic solvent significantly improves the solubility of the analytes in the BGE and allows separation to be performed. This was the situation encountered in the research by Burkinshaw et al. [32]. They wanted to use the MEKC technique, often used to separate neutral compounds, but due to the poor solubility of the analytes in water, standard BGEs were not suitable for analysis. Thus, they decided to add 50% ACN to the background electrolyte. Such approach enabled the separation of the water-insoluble, neutral reactants (dye intermediates) involved in the synthesis reaction of the disperse dye. The composition of the BGE was as follows: 10 mmol·L^−1^ sodium tetraborate, 50 mmol·L^−1^ boric acid, 20 mmol·L^−1^ SDS, and 50% (*v*/*v*) ACN as a co-solvent, pH = 10. Note that, in the mentioned article, there is no actual example of separation of the final products—disperse dyes.

An alternative method to separate disperse dyes is capillary electrochromatography (CEC). The CEC has an advantage over the MEKC, where the employed surfactants are not compatible with the MS detector. In 1995, the possibility of separating disperse dyes using CEC was presented [41]. The mobile phase consisted of ACN mixed with sodium tetraborate at pH = 8 (details in Table 2) was applied. The grains of octadecyl-modified silica gel, the diameter of which was 3 μm, were used as the capillary filling. A mixture of four azo dyes and a mixture of three anthraquinone dyes diluted in the mentioned eluent were analyzed. Thanks to the use of an appropriate capillary filling, it was possible to separate not only the above-mentioned mixtures but also to separate one of the dyes from impurities that were present in the commercially available reagent.

The instrumental conditions used in the above-mentioned examples of disperse dye separation are shown in Table 2.

## 4. Discussion

Among the many groups of textile dyes, some do not require a special approach, as they can be easily analyzed using the simplest CEMs—capillary zone electrophoresis. They are easily soluble in water and aqueous solutions: acid, basic, direct and reactive dyes. Of course, when carrying out optimization, the parameters of the method should be adjusted to the properties and interactions exhibited by a specific group of dyes. It should be remembered that in addition to the standard approaches used (e.g., fused silica capillary), there are other possibilities that may bring favorable results by improving the resolution and selectivity of the method (e.g., amine or neutral capillaries, coated capillaries, etc.).

The neutral dyes (under the given conditions) are slightly more challenging. In this case, techniques that use ionic (anionic or cationic) surfactants, such as MEKC, are a good solution. The most difficult group of dyes are those insoluble (or very slightly soluble) in water: disperse and vat textile dyes.

In the case of the first group, the proposed solution may be a technique using non-aqueous BGE—NACE. Microemulsion electrokinetic capillary chromatography (MEEKC) due to the use of the oil phase also has a high affinity for hydrophobic (lipophilic) substances so might be useful for the separation of such analytes [46,47].

By using the NACE technique, where BGEs are prepared based on organic solvents, it is possible to shorten the total sample preparation time using the same extracting agent as the BGE. This makes it possible to subject the obtained extracts to direct analysis, which reduces the risk of contamination or loss of analytes during the numerous stages of sample preparation. On the other hand, the use of organic solvents increases the costs compared to the analyses carried out in water-based primary electrolytes. In addition, when selecting a solvent, many important parameters should be taken into account. One of them is the cut-off value since most solvents have a high absorption of UV radiation, which can cause loss of analyte signals in the event of spectrophotometric detection. The volatility of the substance is also important, because too volatile solvents may evaporate from the BGE during the measurement or sequence of measurements, which leads to a change in the proportions of the components of the separation buffer and negatively affects the repeatability of the analyses. Due to the high solubility of gases in organic solvents, it is also very important to degass the buffers before the measurement, so that the current circuit is not interrupted, and the detection is not disturbed. It should also be borne in mind that when a mixture of solvents is used as the BGE base, data on the physicochemical properties of these mixtures are not always available in the literature. Additionally, the preparation of BGE based on organic solvents is less compliant with the principles of green chemistry than the use of water buffers.

Considering the amount of organic solvents used to prepare BGE, the MEEKC technique is much more in line with the postulates of green chemistry. Another advantage of using microemulsions as BGE is their stability, lasting up to several months after preparation. The availability of numerous types of surfactants and compounds that can act as oils and co-surfactants gives enormous possibilities in modifying the composition of ME in such a way as to obtain the optimal BGE. On the other hand, it should be noted that the preparation of ME is a time-consuming process.

Taking into account the possibilities offered by modern science, the use of ionic liquids (ILs) as the oil phase seems to be a solution for the future in the case of textile dyes capillary electromigration analysis [47]. First of all, ILs are green solvents that, after appropriate research, can be an alternative to organic solvents used in laboratories so far. In addition, they are characterized by low vapor pressure and high electrochemical stability, so ionic liquid microemulsion (ILME) should not change their composition during the separation process (electrolysis at the electrodes) or long measurement sequences (evaporation). On the other hand, like the organic solvents used in NACE, ILs has a relatively high absorption of radiation in the UV range. Moreover, the price of ionic liquids is relatively high and therefore significantly increases the cost of the analysis.

It should also be noted that most of the scientific articles outlined above-used fibers (or even threads) about 1 cm in length to extract the dyes. The dimensions of the fibers revealed at the scene are much smaller, and therefore, also, the amount of extracted dyes is smaller. Here, methods of concentrating the analytes inside the capillary, which contribute to lowering the detection limit, can be helpful [48,49]. Of course, the mass spectrometer as a detector that significantly increases the sensitivity of the method can be also useful for this purpose [50]. Using MS, however, it is challenging to use the CEMs in which surfactants are included in the BGE composition. The use of volatile surfactants or a partial filling should then be considered.

## 5. Conclusions

In conclusion, a lot has already been performed regarding the study of textile dyes using CEMs. Scientific papers from the 20th century present fast and reliable methods for the separation of water-soluble dyes. However, dyes insoluble in aqueous solutions were and still are problematic. In the 21st century, only a few new scientific reports on the separation of textile dyes by CEMs appeared. There are no articles about capillary electromigration analysis of sulfur dyes. So, it seems that these challenging aspects of separating textile dyes need to be reconsidered, some ideas analyzed again to capture the positive aspects of the approach, but also those not necessarily effective, and then, reintroduced into laboratory development. There seems to be a need and space to do a lot more.

## Figures and Tables

**Table 1:** Groups of dyes and types of fibers dyed by them.

Dye Class	Types of Dyed Fibers				Dye-Fiber Interaction
	Natural		Chemical		
	Plant	Animal	Artificial	Synthetic	
Acid		wool silk	protein	polyamide polyacrylonitrile	ionic bonds, hydrophobic interactions
Basic				polyacrylonitrile polyester polyamide	ionic bonds, hydrophobic interactions
Direct	cotton		viscose		Hydrogen bonds, and Van der Waals forces
Disperse			acetate triacetate	polyester polyacrylonitrile polyamide polypropylene	deposited on fibers
Reactive	cotton	wool		polyamide	covalent bonds
Sulfur	cotton				ionic bonds, Van der Waals forces
Vat	cotton				ionic bonds, Van der Waals forces, and π-H bond

**Table 2:** Condition of detection, injection, separation as well as the composition of background electrolyte (BGE) used in the CEMs dedicated for analysis of textile dyes.

Technique	Detection	Injection	Separation Temperature and Voltage	Capillary	Background Electrolyte	Analyzed Samples	Sample Pre-Treatment	Ref.
**Acid Dyes**								
CZE	UV 254 nm	height difference 5 cm, 10 s	N/A	fused silica, uncoated I.D. = 50 μm L_t_ = 60 cm	10 mmol·L^−1^ KH_2_PO_4_ pH 9	a mixture of 4 commercially available dyes (Acid Red 13, 18, 41, 88) and their intermediates at concentration range 0.15–0.1 g·L^−1^	-	[25]
CZE	UV-Vis 254 nm	hydrodynamic 0.5 psi *, 9 s	22 °C 18 kV	fused silica, uncoated I.D. = 50 μm O.D. = 360 μm L_t_ = 68 cm L_ef_ = 60 cm	0.14 mol·L^−1^ CAPS pH 10.8	dyes extracted from wool (0.25–5.5 cm^2^ material and 2.5 mm–1 cm thread)	extraction was carried out with a mixture of NH_3_:H_2_O (4:3) for 50 min in 100 °C; then 10% (*v*/*v*) of methanol was added to samples, later evaporated and obtained precipitates were dissolved into 300 μL of MeOH:H_2_O (1:1, *v*/*v*) and diluted with water (1:10)	[26]
CZE	UV 214 nm	N/A	25 °C −10 kV	fused silica, polyacryloamide-coated I.D. = 75 μm L_t_ = 26.8 cm L_ef_ = 20.2 cm	0.5% (m/v) PEG 0.01% (m/v) PVP BTP pH 6.5	Natural Blue 2, Acid Yellow 1, Acid Red 2, 27, 26, 88; Acid Orange 7, 12, 52	dyes were dissolved in water at a concentration of 40 ppm	[27]
CZE	DAD 190–600 nm	hydrodynamic 0.2 psi, 2 s	25 °C 30 kV	fused silica, polyimide-coated I.D. = 50 μm O.D. = 75 μm L_t_ = 50 cm L_ef_ = 40 cm	15 mmol·L^−1^ CH_3_COONH_4_ in ACN:H_2_O (40:60, *v*/*v*) pH 9.3	6 samples of acid-dyed nylon 6, standards of the dyes (common in commercial use) with which each fabric was treated 1-cm lengths of the thread containing about 50 fibers	dyes standards were prepared in deionized water at concentration 1 g·L^−1^; extraction was carried out with the use of 400 μL of solvent (50% pyridine 50% ammonia) in 100 °C for 15–30 min; 200 μL of extracts were evaporated to dryness in 50 °C and the residues were dissolved in 100 μL of deionized water	[28]
CZE	DAD 190–600 nm	hydrodynamic 0.2 psi, 2 s	25 °C 30 kV	L_t_ = 50 cm	15 mmol·L^−1^ CH_3_COONH_4_ in ACN:H_2_O (40:60, *v*/*v*) pH 9.3	a mixture of 14 anionic dyes including 3 acid dyes (Acid Blue 45, 239, 277) at a concentration of 1 g·L^−1^; 10 cm nylon fibers	extraction carried out in 66 μL of solvent (mixture consisted of equal parts of aqueous ammonia, pyridine and water) at 100 °C for 60 min, extracts were dried at 60 °C to remove solvents, residuals were dissolved in 190 μL of water	[29]
CZE	ESI-MS	5 kPa	N/A 30 kV	I.D. = 75 μm O.D. = 360 μm L_t_ = 80 cm	0.1 mol·L^−1^ CH_3_COONH_4_ pH 9	Mordant Violet 5, Acid Orange 7, 8	-	[30]
NACE	Cyclic voltammetry UV-Vis 200 nm	height difference 10 cm, 10 s	N/A 20 kV	fused silica, I.D. = 75 μm O.D. = 360 μm	1 mol·L^−1^ CH_3_COOH 10 mmol·L^−1^ CH_3_COONH_4_ in ACN:MeOH (75:25, *v*/*v*)	Acid Green 25, acid red 1, acid blue 324; water samples including river and lake water which were spiked with textile dyes	SPE from aqueous solution-SPE cartridges were activated immediately before use with 3 mL of methanol followed by 6 mL of distilled water, then a sample was applied (flow rate 3 mL·min^−1^), later cartridges were rinsed with 12 mL of distilled water followed by 2 mL of ACN, to elute the dyes 3–5 mL of mixture ACN:THF (2:1, *v*/*v*) was used (flow rate 1 mL·min^−1^)	[31]
MEKC	UV 254 nm	height difference 10 cm, 12 s	N/A 25 kV	fused silica, I.D. = 75 μm L_t_ = 60 cm	10 mmol·L^−1^ Na_2_B_4_O_7_ 40 mmol·L^−1^ SDS	mixture of two acid dyes, concentrations range 30–40 mg·L^−1^	-	[32]
MEKC	DAD 233 nm	hydrodynamic 30–50 mbar, 3 s	20 °C 29 kV	fused silica, I.D. = 50 μm L_t_ = 50 cm	10 mmol·L^−1^ SDS 25 mmol·L^−1^ Na_2_B_4_O_7_ pH 9.3	1 g·L^−1^ aqueous stack solutions of commercially available dyes (Acid Black 194, Acid Brown 432, 434)	dyes solutions diluted 2–5 times before measurement	[33]
**Basic Dyes**								
CZE	UV 254 nm	height difference 50 mm, 5 s	N/A 20 kV	fused silica, uncoated I.D. = 50 μm L_t_ = 60 cm	20 mmol·L^−1^ citric acid pH 4.5	Commercially available dyes (Cartasol Yellow K-GL(S), Astrazon Orange R, Astrazon Red GTLN 200%, Yoracryl Yellow 4G) at a concentration of 0.015–0.2 g·L^−1^	-	[25]
CZE	UV 254 nm	hydrodynamic N/A, 1 s	25 °C 10 kV	fused silica, uncoated I.D. = 75 μm L_t_ = 26.9 cm L_ef_ = 20.2 cm	1% PVP 10 mmol·L^−1^ citric acid TRIS pH 3.0	a mixture of 5 dyes in water including 4 basic dyes (Basic Green 1, Basic Violet 3, Basic Red 9, Basic Blue 9) and 1 acid dye (Acid Red 2) at a concentration of 25 or 100 g·L^−1^	-	[34]
CZE	DAD 190–600 nm	hydrodynamic 0.2 psi, 2 s	25 °C 20 kV	fused silica polyimide-coated I.D. = 50 μm L_t_ = 50 cm L_ef_ = 40 cm	45 mmol·L^−1^ CH_3_COONH_4_ in ACN:H_2_O (60:40, *v*/*v*) pH 4.7 adjusting with CH_3_COOH	1 cm acrylic thread containing 30–50 fibers	extraction was carried out with use 200 μL of solvent (mixture of 88% formic acid and 12% water) at 100 °C for 60 min, after evaporation to dryness in 50 °C, the extract was reconstituted with 190 μL of water	[35]
	ESI-QTOF-MS	hydrodynamic 1 psi, 5 s				2 mm acrylic single fiber	extraction was carried out with use 10 μL of solvent (mixture of 88% formic acid and 12% water) at 100 °C for 60 min, after evaporation to dryness, the extract was reconstituted with 5 μL of water	
NACE	cyclic voltammetry UV-Vis 200 nm	height difference 10 cm, 10 s	N/A 20 kV	fused silica I.D. = 75 μm O.D. = 360 μm	1 mol·L^−1^ CH_3_COOH 10 mmol·L^−1^ CH_3_COONH_4_ in ACN:MeOH (75:25, *v*/*v*)	Basic Blue 9, 41, Basic Violet 3, 16; Basic Green 4; water samples including river and lake water which were spiked with textile dyes	SPE from aqueous solution-SPE cartridges were activated before use with 3 mL of methanol followed by 6 mL of distilled water, then a sample was applied (flow rate 3 mL·min^−1^), later cartridges were rinsed with 12 mL of distilled water followed by 2 mL of ACN, to elute the dyes 3–5 mL of mixture ACN:THF (2:1, *v*/*v*) was used (flow rate 1 mL·min^−1^)	[31]
**Reactive Dyes**								
CZE	UV-Vis 520 nm	height difference 10 cm, 25 s	N/A	fused silica, uncoated I.D. = 50 μm L_t_ = 60 cm	10 mmol·L^−1^ Na_2_B_4_O_7_ 50 mmol·L^−1^ H_3_BO_3_ pH 10 adjusted with NaOH	a mixture of commercially available reactive dyes and their derivatives at a concentration of 0.1 g·L^−1^	-	[25]
CZE	UV	N/A	N/A 20 kV	fused silica, uncoated I.D. = 75 μm L_t_ = 57 cm	10 mmol·L^−1^ Na_2_B_4_O_7_ 6 mmol·L^−1^ KH_2_PO_4_ pH 8.25	Reactive Red 120, 141; Reactive Blue 171, Reactive Yellow 84, Reactive Green 19;	hydrolysis (10 mg of dye mixed with 700 mg of sodium sulphate and dissolved in water, a solution placed in a water bath set as 95 °C for 20 min, then 200 mg sodium carbonate was added to the mixture—pH 10.5—and placed in the water bath at 80 °C for one hour); to remove the salts samples were subjected to dialysis and then analyzed	[36]
CZE	UV-Vis 254 nm	hydrodynamic 0.5 psi, 9 s	22 °C 18 kV	fused silica, uncoated I.D. = 50 μm O.D. = 360 μm L_t_ = 68 cm L_ef_ = 60 cm	0.14 mol·L^−1^ CAPS pH 10.8	cotton material (0.25–5.5 cm^2^)	extraction was carried out with the use 1.5% NaOH for 25 min at 100 °C; 10% (*v*/*v*) of methanol was added to samples, then evaporated, obtained precipitates were dissolved into 300 μL of MeOH:H_2_O (1:1, *v*/*v*) and diluted with water (1:10)	[26]
CZE	UV-Vis 608 nm	hydrodynamic 30 s	N/A	fused silica I.D. = 75 μm L_t_ = 70 cm L_ef_ = 63 cm	20 mmol·L^−1^ Na_2_B_4_O_7_ 10% ACN pH 9.26	Malachite Green, Indygo Carmine, Cibacron Blue, Remazol Black B	dyes were hydrolyzed on a water bath for 10 min at 60–70 °C using 10^−3^ mol·L^−1^ sodium hydroxide, hydrolyzed dyes solutions in water were tested, concentration range 10^−6^–10^−8^ mol·L^−1^	[37]
CZE	DAD 190–600 nm	hydrodynamic 50 mbar, 2 s	25 °C 20 kV	fused silica I.D. = 75 μm L_t_ = 50 cm L_ef_ = 42 cm	5 mmol·L^−1^ CH_3_COONH_4_ in ACN:H_2_O (40:60) pH 9.3	12 commercially available dyes (Reactive Blue 19, 21, 220, 250; Reactive Yellow 160, 176; Reactive Orange 72, Reactive Violet 5, Reactive Black 5, Reactive Red 180, 198, 239, 241) concentrations 0.1 mg·mL^−1^; 10 cm cotton multifilament thread	extraction of dyes with alkaline hydrolysis (0.2 mL of 1.5% aqueous sodium hydroxide), SPE for extracts cleanup (conditioning of cartridges with 1 mL of methanol followed by 1 mL of water, applying of extracts, rinsing with 1 mL of 5% methanol in water, eluting dyes with 1–2 mL of methanol), dyes recovered from SPE were reconstituted in 0.1–0.2 mL of water and analyzed	[38]
RP-CE	DAD 400–610 nm	hydrodynamic	25 °C −25 kV	fused silica I.D. = 75 μm L_t_ = 75 cm L_ef_ = 68 cm	0.05 mol·L^−1^ citric acid pH 3.25 10% ACN (*v*/*v*)	Remazol Black B, Remazol Red RB (solutions were prepared from solid dyes), Remazol Navy Blue GG, Remazol Golden Yellow RNL, Cibacron Red C-2G (33% water-sulfuric acid solution, 67 g of dye per 100 mL of solution)	hydrolysis carried out in water-bath heated to 60–70 °C, equal volumes of dye and 0.001 mol·L^−1^ NaOH were mixed and stirred for 10 min	[39]
**Vat Dyes**								
CZE	DAD 190–600 nm	hydrodynamic 50 mbar, 2 s	25 °C 20 kV	fused silica I.D. = 75 μm L_t_ = 50 cm L_ef_ = 42 cm	5 mmol·L^−1^ CH_3_COONH_4_ 20 mmol·L^−1^ Na_2_S_2_O_4_ in ACN:H_2_O (40:60) pH 9.3	mixture of 3 commercially available dye (Vat Yellow 2, Vat Orange 2, Vat Black 16) at a concentration: 1g·L^−1^; 10 cm cotton thread	for extraction reduction was performed using a reducing agent (sodium dithionite dissolved in 1,2-dimethoxy ethane), dyes recovered from extraction were reconstituted in 200 μL of water with dithionite and analyzed	[38]
CZE	ESI-MS	height difference 10 cm, 5 s	N/A 25 kV	fused silica, uncoated I.D. = 75 μm L_t_ = 120 cm	1 mmol·L^−1^ CH_3_COONH_4_ pH 10 12.5% ACN	a mixture of 3 sulfonated derivatives of vat dyes (Vat Blue 1, 2, 5) at a concentration of 500 nmol·mL^−1^ in water	-	[40]
**Direct Dyes**								
CZE	DAD 190–600 nm	hydrodynamic 50 mbar, 2 s	25 °C 20 kV	fused silica I.D. = 75 μm L_t_ = 50 cm L_ef_ = 42 cm	5 mmol·L^−1^ CH_3_COONH_4_ in ACN:H_2_O (40:60) pH 9.3	mixture of 7 commercially available direct dyes (Direct Black 23, 112; Direct Red 84, Direct Orange 39, Direct Yellow 58, 86; Direct Blue 71) at a concentration of 1 g·L^−1^; 10 cm of multifilament cotton threads,	extraction of Direct Yellow 58 from cotton fibers was carried out with the use of 0.2 mL solvent mixture (35% pyridine-65% water) at 60 °C for 60 min, then the solvent was evaporated to dryness in 50 °C, dyes recovered from extraction were reconstituted in 0.2 mL of water and analyzed	[38]
CZE	DAD 190–600 nm	hydrodynamic 0.2 psi, 2 s	25 °C 30 kV	L_t_ = 50 cm	15 mmol·L^−1^ CH_3_COONH_4_ in ACN:H_2_O (40:60, *v*/*v*) pH 9.3	a mixture of five acid, four direct and five reactive dyes at concentration of 1 g·L^−1^	-	[29]
**Disperse dyes**								
NACE	DAD 190–600 nm	hydrodynamic 0.2 psi, 2 s	25 °C 30 kV	L_t_ = 50 cm	80 mmol·L^−1^ CH_3_COONH_4_ in ACN:MeOH (75:25, *v*/*v*) pH* 9	1 cm polyester fiber (obtained electropherogram not shown)	extraction was carried out with use 0.2 mL of chlorobenzene at 100 °C for 60 min	[29]
MEKC	UV 254 nm	height difference 10 cm, 12 s	N/A 25 kV	fused silica, uncoated I.D. = 75 μm L_t_ = 60 cm	10 mmol·L^−1^ Na_2_B_4_O_7_ 50 mmol·L^−1^ H_3_BO_3_ 20 mmol·L^−1^ SDS 50% (*v*/*v*) ACN	standard solutions of intermediates appearing during synthesis process of disperse dye; concentration range: 20–100 mg·L^−1^	-	[32]
CEC	UV 210 nm	electrodynamic	N/A 30 kV	fused silica, uncoated I.D. = 75 μm O.D. = 375 μm L_t_ = 100 cm	ACN 4 mmol·L^−1^ Na_2_B_4_O_7_ (80:20, *v*/*v*) pH 8 adjusted with NaOH	commercially available azo dyes: Serilene orange 2RL, Serilene Dark Red FL, Serilene Yellow Brown R-LS, Serilene Yellow Brown G-LS; antraquinone dyes: Dispersol Blue BN, Dispersol Red B3B, Terasil Blue 2R	samples were diluted in the eluent	[41]
	ESI-MS							

I.D.—capillary internal diameter; O.D.—capillary outer diameter; L_t_—capillary total length; L_ef_—capillary effective length; N/A—data not available; *—1 psi = 6894.76 Pa; CZE—capillary zone electrophoresis; MEKC—micellar electrokinetic capillary electrophoresis; NACE—nonaqueous capillary electrophoresis; RP-CE—reverse polarity capillary electrophoresis; CEC—capillary electrochromatography.

## Data Availability

Not applicable.

## References

[1] Wąs-Gubała J., Starczak R. (2015). Nondestructive identification of dye mixtures in polyester and cotton fibers using Raman spectroscopy and ultraviolet–visible (UV-Vis) Microspectrophotometry. Appl. Spectrosc..

[2] Wąs-Gubała J., Brożek-Mucha Z. (2009). Forensic examinations of clothing and other materials found in the coffin by the body of general Władysław Sikorski. Arch. Med. Sadowej Kryminol..

[3] Wąs-Gubała J., Siegel J.A., Saukko P.J., Houck M.M. (2013). Textile and fiber damage. Encyclopedia of Forensic Sciences.

[4] Goodpaster J.V., Liszewski E.A. (2009). Forensic analysis of dyed textile fibers. Anal. Bioanal. Chem..

[5] Kadolph S.J. (2017). , Marcketti, S.B. Textiles.

[6] Śmigiel-Kamińska D., Pośpiech J., Makowska J., Stepnowski P., Wąs-Gubała J., Kumirska J. (2019). The identification of polyester fibers dyed with disperse dyes for forensic purposes. Molecules.

[7] (2020). Preferred Fiber and Materials Market Report. https://textileexchange.org/2020-preferred-fiber-and-materials-market-report-pfmr-released-2.

[8] Lewis S., Houck M.M. (2009). Analysis of dyes using chromatography. Identification of Textile Fibers.

[9] Color Index. https://colour-index.com.

[10] Tehrani A., Holmberg K. (2013). Solubilization of hydrophobic dyes in surfactant solutions. Materials.

[11] Shore J. (1990). Colorants and Auxiliaries. Organic Chemistry and Application Properties.

[12] Tello-Burgos N., López-Montes A.M., Ballesta-Claver J., Collado-Montero F.J., García M.D.R.B. (2021). Identification of Indigo dye (Indigofera tinctoria) and its degradation products by separation and spectroscopic techniques on historic documents and textile fibers. Stud. Conserv..

[13] Śmigiel-Kamińska D., Pośpiech J., Stepnowski P., Wąs-Gubała J., Kumirska J. (2021). Development of HPLC-DAD and UPLC-QTOF-MS chromatographic systems for the identification for forensic purposes of disperse dyes of polyester. J. Meas. Confed..

[14] Beldean-Galea M.S., Copaciu F.-M., Coman M.-V. (2018). Chromatographic analysis of textile dyes. J. AOAC Int..

[15] Shahid M., Wertz J., Degano I., Aceto M., Khan M.I., Quye A. (2019). Analytical methods for determination of anthraquinone dyes in historical textiles: A review. Anal. Chim. Acta.

[16] Carey A., Rodewijk N., Xu X., van der Weerd J. (2013). Identification of dyes on single textile fibers by HPLC-DAD-MS. Anal. Chem..

[17] Schotman T.G., Xu X., Rodewijk N., van der Weerd J. (2017). Application of dye analysis in forensic fibre and textile examination: Case examples. Forensic Sci. Int..

[18] Hu C., Zhu J., Mei H., Shi H., Guo H., Zhang G., Wang P., Lu L., Zheng X. (2018). A sensitive HPLC-MS/MS method for the analysis of fiber dyes. Forensic Chem..

[19] Vasileiadou A., Karapanagiotis I., Zotou A. (2021). Development and validation of a liquid chromatographic method with diode array detection for the determination of anthraquinones, flavonoids and other natural dyes in aged silk. J. Chromatogr. A.

[20] Śmigiel-Kamińska D., Jolanta Wąs-Gubała J., Piotr Stepnowski P., Kumirska J. (2020). The identification of cotton fibers dyed with reactive dyes for forensic purposes. Molecules.

[21] Farah S., Kunduru K.R., Tsach T., Bentolila A., Domb A.J. (2015). Forensic comparison of synthetic fibers. Polym. Adv. Technol..

[22] Lepot L., Lunstroot K., De Wael K. (2020). Interpol review of fibres and textiles 2016–2019. Forensic Sci. Int. Synerg..

[23] Gao Z., Zhong W. (2022). Recent (2018–2020) development in capillary electrophoresis. Anal. Bioanal. Chem..

[24] Voeten R.L.C., Ventouri I.K., Haselberg R., Somsen G.W. (2018). Capillary electrophoresis: Trends and recent advances. Anal. Chem..

[25] Croft S.N., Hinks D. (1992). Analysis of dyes by capillary electrophoresis. J. Soc. Dye. Colour..

[26] Sirén H., Sulkava R. (1995). Determination of black dyes from cotton and wool fibres by capillary zone electrophoresis with UV detection: Application of marker technique. J. Chromatogr. A.

[27] Blatny P., Fischer C.-H., Rizzi A., Kenndler E. (1995). Linear polymers applied as pseudo-phases in capillary zone electrophoresis of azo compounds used as textile dyes. J. Chromatogr. A.

[28] Stefan A.R., Dockery C.R., Nieuwland A.A., Roberson S.N., Baguley B.M., Hendrix J.E., Morgan S.L. (2009). Forensic analysis of anthraquinone, azo, and metal complex acid dyes from nylon fibers by micro-extraction and capillary electrophoresis. Anal. Bioanal. Chem..

[29] Morgan S.L., Vann B.C., Baguley B.M., Stefan A.R. (2007). Advances in discrimination of dyed textile fibers using capillary electrophoresis/mass spectrometry. https://projects.nfstc.org/trace/docs/Trace%20Presentations%20CD-2/morgan_dyed_textiles.pdf.

[30] Lu Y., Phillips D.R., Lu L., Hardin I.R. (2008). Determination of the degradation products of selected sulfonated phenylazonaphthol dyes treated by white rot fungus Pleurotus ostreatus by capillary electrophoresis coupled with electrospray ionization ion trap mass spectrometry. J. Chromatogr. A.

[31] Pelaezcid A., Blascosancho S., Matysik F. (2008). Determination of textile dyes by means of non-aqueous capillary electrophoresis with electrochemical detection. Talanta.

[32] Burkinshaw S.M., Hinks D., Lewis D.M. (1993). Capillary zone electrophoresis in the analysis of dyes and other compounds employed in the dye-manufacturing and dye-using industries. J. Chromatogr. A.

[33] Sebastiano R., Contiello N., Senatore S., Righetti P.G., Citterio A. (2012). Analysis of commercial Acid Black 194 and related dyes by micellar electrokinetic chromatography. Dye. Pigment..

[34] Blatny P., Fischer C.-H., Kenndler E. (1995). Improvement of the separation selectivity in capillary zone electrophoresis of synthetic cationic dyes by polyvinylpyrrolidone as pseudo-stationary phase. Fresenius. J. Anal. Chem..

[35] Stefan A.R., Dockery C.R., Baguley B.M., Vann B.C., Nieuwland A.A., Hendrix J.E., Morgan S.L. (2009). Microextraction, capillary electrophoresis, and mass spectrometry for forensic analysis of azo and methine basic dyes from acrylic fibers. Anal. Bioanal. Chem..

[36] Hansa A., Pillay V.L., Buckley C.A. (1999). Analysis of reactive dyes using high performence capillary electrophoresis. Water Sci. Technol..

[37] Farry L., Oxspring D.A., Smyth W., Marchant R. (1997). A study of the effects of injection mode, on-capillary stacking and off-line concentration on the capillary electrophoresis limits of detection for four structural types of industrial dyes. Anal. Chim. Acta.

[38] Dockery C.R., Stefan A.R., Nieuwland A.A., Roberson S.N., Baguley B.M., Hendrix J.E., Morgan S.L. (2009). Automated extraction of direct, reactive, and vat dyes from cellulosic fibers for forensic analysis by capillary electrophoresis. Anal. Bioanal. Chem..

[39] Oxspring D.A., Smyth W.F., Marchant R. (1995). Comparison of reversed-polarity capillary electrophoresis and adsorptive stripping voltammetry for the detection and determination of reactive textile dyes. Analyst.

[40] Tetler L.W., Cooper P.A., Carr C.M. (1994). The application of capillary electrophoresis/mass spectrometry using negative-ion electrospray ionization to areas of importance in the textile industry. Rapid Commun. Mass Spectrom..

[41] Lord G., Gordon D., Tetler L., Carr C. (1995). Electrochromatography-electrospray mass spectrometry of textile dyes. J. Chromatogr. A.

[42] Shu D., Fang K., Liu X., Cai Y., An F. (2020). High dye fixation pad-steam dyeing of cotton fabrics with reactive dyes based on hydrophobic effect. J. Nat. Fibers.

[43] Chen W., Gao P., Jiang H., Cui Z. (2019). A novel reactive dyeing method for silk fibroin with aromatic primary amine-containing dyes based on the Mannich reaction. Dye. Pigment..

[44] Lucio D., Laurent D., Roger G. (2008). Adsorption of Remazol Black B dye on activated carbon felt. Carbon Sci. Tech..

[45] Benkhaya S., El Harfi S., El Harfi A. (2017). Classifications, properties and applications of textile dyes: A review. Appl. J. Environ. Eng. Sci..

[46] Yu L., Chu K., Ye H., Liu X., Xu X., Chen G. (2012). Recent advances in microemulsion electrokinetic chromatography. TrAC Trends Anal. Chem..

[47] Yang H., Ding Y., Cao J., Li P. (2013). Twenty-one years of microemulsion electrokinetic chromatography (1991–2012): A powerful analytical tool. Electrophoresis.

[48] Xu X., Leijenhorst H., Hoven P.V.D., De Koeijer J., Logtenberg H. (2001). Analysis of single textile fibres by sample-induced isotachophoresis-micellar electrokinetic capillary chromatography. Sci. Justice J. Forensic Sci. Soc..

[49] Šlampová A., Malá Z., Gebauer P., Boček P. (2017). Recent progress of sample stacking in capillary electrophoresis (2014–2016). Electrophoresis.

[50] Breadmore M.C., Grochocki W., Kalsoom U., Alves M.N., Phung S.C., Rokh M.T., Cabot J.M., Ghiasvand A., Li F., Shallan A.I. (2019). Recent advances in enhancing the sensitivity of electrophoresis and electrochromatography in capillaries and microchips (2016–2018). Electrophoresis.

